# CoRRE Trait Data: A dataset of 17 categorical and continuous traits for 4079 grassland species worldwide

**DOI:** 10.1038/s41597-024-03637-x

**Published:** 2024-07-18

**Authors:** Kimberly J. Komatsu, Meghan L. Avolio, Josep Padullés Cubino, Franziska Schrodt, Harald Auge, Jeannine Cavender-Bares, Adam T. Clark, Habacuc Flores-Moreno, Emily Grman, W. Stanley Harpole, Jens Kattge, Kaitlin Kimmel, Sally E. Koerner, Lotte Korell, J. Adam Langley, Tamara Münkemüller, Timothy Ohlert, Renske E. Onstein, Christiane Roscher, Nadejda A. Soudzilovskaia, Benton N. Taylor, Leho Tedersoo, Rosalie S. Terry, Kevin Wilcox

**Affiliations:** 1 Department of Biology, University of North Carolina at Greensboro, Greensboro, NC USA; 2 Department of Earth and Planetary Sciences, Johns Hopkins University, Baltimore, MD USA; 3 Centre for Research on Ecology and Forestry Applications (CREAF), Barcelona, Spain; 4 School of Geography, University of Nottingham, Nottingham, UK; 5 UFZ, Helmholtz Centre for Environmental Research, Community Ecology, Theodor-Lieser-Strasse 4, 06120 Halle, Germany; 6 German Centre for Integrative Biodiversity Research (iDiv) Halle-Jena-Leipzig, Puschstrasse 4, 04103 Leipzig, Germany; 7 Department of Ecology, Evolution and Behaviour, University of Minnesota, Saint Paul, MN USA; 8 University of Graz, Institute of Biology, Holteigasse 6, 8010 Graz, Austria; 9 CSIRO Health and Biosecurity, GPO Box 2583, Brisbane, QLD 4001 Australia; 10 Department of Biology, Eastern Michigan University, Ypsilanti, MI USA; 11 UFZ, Helmholtz Centre for Environmental Research, Physiological Diversity, Permoserstrasse 15, 04318 Leipzig, Germany; 12 Martin Luther University Halle-Wittenberg, Halle (Saale), Germany; 13 Max Planck Institute for Biogeochemistry, Jena, Germany; 14 Mad Agriculture, Boulder, CO USA; 15 Department of Biology, Center for Biodiversity and Ecosystem Stewardship, Villanova University, Villanova, PA USA; 16 Univ. Grenoble Alpes, Univ. Savoie Mont Blanc, CNRS, LECA, Grenoble, France; 17 Department of Biology, Colorado State University, Fort Collins, CO USA; 18 Naturalis Biodiversity Center, Leiden, Netherlands; 19 Centre for Environmental Sciences (CMK), Hasselt University, Hasselt, Belgium; 20 Department of Organismic and Evolutionary Biology, Harvard University, Cambridge, MA USA; 21 Mycology and Microbiology Center, University of Tartu, Tartu, Estonia

**Keywords:** Community ecology, Biodiversity, Plant ecology

## Abstract

In our changing world, understanding plant community responses to global change drivers is critical for predicting future ecosystem composition and function. Plant functional traits promise to be a key predictive tool for many ecosystems, including grasslands; however, their use requires both complete plant community and functional trait data. Yet, representation of these data in global databases is sparse, particularly beyond a handful of most used traits and common species. Here we present the CoRRE Trait Data, spanning 17 traits (9 categorical, 8 continuous) anticipated to predict species’ responses to global change for 4,079 vascular plant species across 173 plant families present in 390 grassland experiments from around the world. The dataset contains complete categorical trait records for all 4,079 plant species obtained from a comprehensive literature search, as well as nearly complete coverage (99.97%) of imputed continuous trait values for a subset of 2,927 plant species. These data will shed light on mechanisms underlying population, community, and ecosystem responses to global change in grasslands worldwide.

## Background & Summary

Ecologists are tasked with forecasting community and ecosystem responses to global change drivers. Functional traits have been put forward as a “holy grail” approach capable of generalizing the link between community and functional processes across scales^1–3^. Plant functional traits — characteristics or measures that indirectly impact the fitness of an individual^4^ — are known to influence species tolerances to environmental conditions^5–7^, competitive outcomes^8–10^, trophic interactions^11^, and ultimately species abundances^12–15^. Additionally, scaling plant functional traits to the community-level by integrating species trait values with their abundances (*i.e*., community-weighted traits) can illuminate community responses to environmental drivers^3,16,17^, as well as enable us to predict the effects of traits on ecosystem processes^1,3,18–20^ across a wide array of ecosystems^21^.

Grasslands and other herbaceous ecosystems are globally important pools of biodiversity and are critical for the sustained provisioning of ecosystem services^22–25^. Yet our global grasslands are under threat due to increased human activities, making understanding the trait-based mechanisms underlying their community assembly and ecosystem function more imperative than ever^26^. Experiments in which global change drivers are manipulated and community and ecosystem response data are collected are one powerful tool to understand and predict grassland responses to global change factors^27^. In order to utilize a trait-based approach to synthesizing grassland responses, it is necessary to gather complete data across all species for traits expected to respond to global change manipulations and/or drive subsequent ecosystem responses^17,28–30^.

Collecting the necessary plant community composition and trait data is time and labour intensive. While databases of plant community composition in response to experimental manipulations in herbaceous ecosystems have begun to emerge^31–33^, complete trait data for an entire plant community is more difficult to obtain. In particular, some plant traits are notoriously difficult to measure and data are consequently sparse (*e.g*., many belowground plant traits^34^). Yet even the plant traits that are relatively easy to measure, such as specific leaf area and leaf dry matter content^35,36^, tend to be available only for the most abundant species in common ecosystem types. Additionally, information for many categorical traits is dispersed across the literature and may not align with the same definitions across sources.

To meet data demands, trait databases have been developed that bring together a global community of contributors and users, including the TRY^37^ and BIEN^38,39^ global databases, as well as many regional trait databases. Yet, despite the impressive amount of plant trait data amassed by the ecological community to date, there remain critical gaps in available data for many species and traits. Many trait-based statistical approaches require complete datasets, which means there can be no missing data across the species and traits investigated^40,41^. Thus, it becomes necessary to impute trait values for species with missing data^42–44^ or extrapolate from close phylogenetic relatives^45^ to generate the complete plant trait databases that are critical for downstream analyses^46,47^. However, imputation methods are typically only used for continuous trait data and are only as powerful as the trait data being fed into them, resulting in both significant remaining missing data and potentially inaccurate data. They also have the potential to give rise to circular analyses, for example when evolutionary processes are investigated using traits imputed using phylogenetic information.

**Fig. 1:**
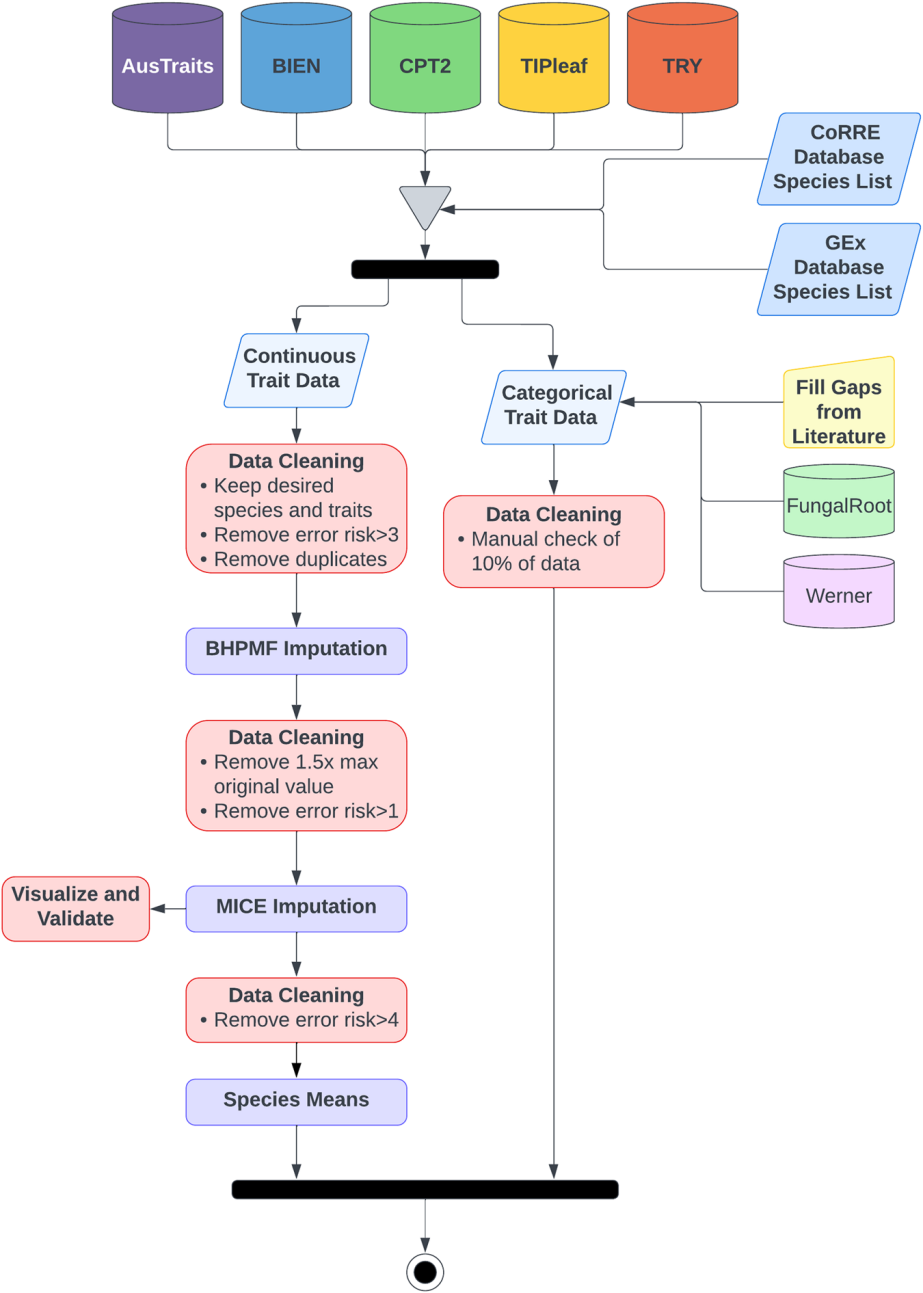
Flowchart of trait data generation, including gathering existing data from five plant trait databases for vascular plant species within the CoRRE and GEx databases, gap filling through imputation (continuous traits) or from the literature (categorical traits), and data cleaning at each step.

A pressing need in ecology is to determine how plant functional traits determine or are mechanistically associated with species’ responses to global change in grassland ecosystems around the world. Towards that end, we aim to bridge the gap between existing databases that have assembled plant community and trait information and the complete data we require. To do so, we have identified the gaps in existing data sources and filled those gaps with data from an intensive literature search following clear data gathering protocols (categorical traits) and statistical imputation methods based on a set of measured trait data from existing plant trait databases (continuous traits). This effort has resulted in a unique and nearly complete trait dataset^48^ comprised of (1) a suite of 9 categorical traits (Table 1) for all 4,079 vascular plant species across 173 families found within 138 experiments from the Community Responses to Resource Experiments (CoRRE) database (https://corredata.weebly.com/) and 252 experiments from the Grazing Exclosure (GEx) database (https://koernerlab.weebly.com/) and (2) 8 continuous traits (Table 2) for a subset of 2,927 of these same vascular plant species across 147 plant families (Fig. 1). These traits were selected to encompass those that were expected to meaningfully contribute to plant species responses to global change drivers or effects on ecosystem function, within the limitations of data availability (see methods below). The assembled trait dataset^48^ will allow us to directly link complete data on plant community responses to global change drivers to the traits of these species and ultimately their ecosystem outcomes.

**Table 1:** Description of categorical traits included in this dataset.

Trait Name [Trait Code]	Categories
Growth Form [growth_form]	graminoid, forb, fern, cactus, vine, lycophyte, woody
Lifespan [lifespan]	annual, biennial, perennial
Clonal [clonal]	yes, no, uncertain
Leaf Type [leaf_type]	broad, narrow, needle, scale, frond, microphyll, modified, none
Leaf Compoundness [leaf_compoundness]	simple, compound, none
Stem Support [stem_support]	self-supporting, pendent, epiphyte, decumbent, prostrate, climbing
Photosynthetic Pathway [photosynthetic_pathway]	C_3_, C_4_, CAM, hybrid, parasitic, uncertain
Mycorrhizal Type [mycorrhizal_type]	AM (arbuscular mycorrhizae), EcM (ectomycorrhizae), ErM (ericaceous mycorrhizae), OM (orchidaceous mycorrhizae), multiple, none, uncertain
Nitrogen Fixation Type [n_fixation_type]	none, rhizobial, actinorhizal

See Supplemental File 1 for a complete description of each trait category.

**Table 2:** Description of continuous traits included in this dataset.

Trait Name [Trait Code]	Description [imputed range]	Units
Vegetative Height [plant_height_vegetative]	Height of vegetative growth [0.007–20.957]	m
Leaf Area (leaf, +petiole) [leaf_area]	Leaf area of leaf, including petiole [0.050–61,213]	mm^2^
*Leaf Area (leaflet, -petiole)*	*Leaf area of leaflet, excluding petiole*	*mm*^2^
*Leaf Area (undefined, undefined)*	*Leaf area, undefined if leaf/leaflet and petiole included/excluded*	*mm*^2^
Leaf Dry Mass [leaf_dry_mass]	Dry mass of a single leaf [0.001–4,292]	mg
Leaf Dry Matter Content [LDMC]	Leaf dry mass per leaf fresh mass [0.002–0.997]	g g^−1^
Specific Leaf Area (+petiole) [SLA]	Leaf area per leaf dry mass, including petiole [1.125–124.160]	mm^2^ mg^−1^
*Specific Leaf Area (-petiole)*	*Leaf area per leaf dry mass, excluding petiole*	*mm*^2^ *mg*^*−1*^
*Specific Leaf Area (undefined)*	*Leaf area per leaf dry mass, undefined if petiole included/excluded*	*mm*^2^ *mg*^*−1*^
Leaf N [leaf_N]	Leaf nitrogen content per leaf dry mass [5.792–61.708]	mg g^−1^
Specific Root Length (all root) [SRL]	Root length per root dry mass of all roots [0.450–312,733]	cm g^−1^
*Specific Root Length (fine root)*	*Root length per root dry mass of fine roots only*	*cm g*^*−1*^
Seed Dry Mass [seed_dry_mass]	Seed dry mass [0.001–171.432]	mg

Traits in italics were utilized for continuous trait imputation, but are not presented in the final dataset. Range of imputed trait means are included in brackets adjacent to each description.

### Summary

Throughout the process of assembling the CoRRE Trait Data^48^, we learned four important lessons that we wish to pass on to the ecological community.

1. The trade-offs among continuous trait data completeness (including the number and distribution of individual measurements for each species), size of the observed data matrix, and the number of traits being imputed may limit the scope and quality of the imputed trait dataset. Originally, we had hoped to include many more than 8 continuous traits in the published dataset (*e.g*., physiological traits, root traits, reproductive traits), however with particularly sparse data we determined it is better to retain only traits with the highest data coverage. We found that inclusion of traits with extremely sparse data (in our case, less than 10% of plant individuals with at least one value for each trait) resulted in a matrix with many times more missing data that would need to be imputed. That is, the inclusion of sparse coverage traits resulted in a non-linear increase in missing data, thereby decreasing the quality of the final imputed dataset. We learned that inclusion of a fewer number of data-rich traits for imputation is advisable. In our case, we included a suite of the most commonly available traits for our species of interest, but also included one additional trait (specific root length) that had few records, yet was both ecologically important and occupied a unique trait dimension (*i.e*., not highly correlated with other trait values), warranting inclusion in downstream analyses.
2. Data imputation methods are only as robust as the measured data that are passed to them. Some of the trait databases on which the ecological community currently relies contain data that may not be appropriate for a given analysis (*e.g*., data from juvenile plants or from experimental conditions like glasshouses or climate chambers), data that may be inaccurate due to discrepancies in measurement methods across investigators, and/or repeated data, which can affect the results of both trait imputation and statistical analyses of traits as drivers of community and ecosystem dynamics. In particular, while they are highly valuable contributions to the ecological community as the primary source of the vast majority of plant trait data, the TRY^37^ and BIEN^38,39^ databases contain data that should be carefully examined prior to any analysis. Despite the massive cleaning and harmonization efforts undertaken to produce TRY and BIEN, we found in some cases that inaccurate data were included in the database and needed to be removed prior to analysis, primarily when units or methods were not standardized to the database convention. In addition, we identified three ways in which trait data were repeated within the TRY database, which can result in inflated confidence in a given trait value both within and across species. First, a dataset may contain multiple measurements of a given trait for each Observation ID (which in the ideal case is meant to be a unique identifier for a plant individual) because multiple leaves were measured for that individual. Because there is no way to link different trait measurements to these individual leaves from a plant within TRY, we averaged data by Observation ID in these cases. Second, a dataset may contain multiple measurements of a given trait for each Observation ID because multiple measurements were made through time (*e.g*., measuring plant height multiple times over a growing season). While in some cases a temporal identifier was provided by the investigator, in many others this was not the case. When temporal data were identified, we took either the mean or maximum value for each Observation ID, depending on the nature of the trait. Finally, truly repeated data were found within TRY, where the same value to an accuracy of five or more decimal places was found across many Dataset and Observation IDs for a given species. In these cases, it seems likely that the same data was entered into TRY multiple times and we used a single value to prevent over-representation of that data in the overall dataset. Although some duplicate entries are flagged in TRY (primarily across DatasetID), in many other cases the reason for repeated data often had to be inferred from the values of the observations and were thus difficult to detect.
3. It is important to carefully consider the data that results from continuous trait imputation. While our imputed data exhibited a similar distribution to the measured data overall, some extreme outliers were generated during the imputation process. Removal of these outliers using standard practices (*e.g*., considering error risks) is relatively straightforward. However, any individual datapoint should be considered carefully based on expert knowledge of each species and trait prior to use in further analyses, as incorrect values can hide within the bulk of the data for any given trait and species.
4. Finally, it is possible to develop complete categorical trait datasets for the most common plant traits (Table 1) through an exhaustive manual search of the literature, online floras, and other online resources. This is possible for categorical traits (compared to continuous traits) due to the fixed nature of categorical traits (*e.g*., a plant’s photosynthetic pathway does not differ depending on location, study, or measurement methods), so that a species’ categorical trait value can be identified from a small subset of studies. To collect our categorical trait data, we learned that it is important to develop standardized methods to ensure accurate data collection and to conduct error checks to determine data accuracy. However, these efforts may not be possible for rarely studied traits, as was the case in our failed efforts to collect complete data for pollination and dispersal modes. Nonetheless, with the rise of machine learning and other algorithms trained on large data inputs, our ability to create datasets of less common categorical traits for many species will likely become increasingly achievable.

## Methods

The existing CoRRE^32^ and GEx^31^ databases contain plant community composition data from individual experiments in herbaceous ecosystems around the world. Here we present the new CoRRE Trait Data^48^, a dataset of traits for all vascular plant species (to the extent possible) within the original CoRRE and GEx databases (Fig. 1). Requirements for inclusion of an experiment in the CoRRE database are that the experiment is located within a grassland ecosystem (*i.e*., herbaceous), directly manipulates a resource (soil nutrients, water, atmospheric CO_2_, and/or light), has at least 3 years of continuous experimental treatments and at least 4 replicates, and has species abundance data^32^. Requirements for inclusion of an experiment in the GEx database are that experiments were located in a grassland ecosystem, have paired plots that are ungrazed vs grazed by large herbivores, had fences in place for a minimum of three years, and have species abundance data^31^. Other than their use for determining which vascular plant species to focus on, no other data from the CoRRE and GEx databases were utilized during the creation of the CoRRE Trait Data^48^.

We standardized species names for all plant species represented in the CoRRE and GEx databases to ‘The Plant List’ using the TaxonStand version 2.4 package in R^49^. Trees and non-vascular plants (*e.g*., mosses) were removed from the dataset. Additionally, plants whose names did not provide taxonomic resolution at the species level (*e.g*., *Aster* sp. or “unk fuzzy plant”) were removed from the dataset. Finally, any species whose names did not result in a match from TaxonStand were cleaned by hand using the World Flora Online^50^.

### Continuous trait data cleaning

For every species, data were pulled from the TRY Plant Trait Database version 6.0^37^ (accessed May 2023), AusTraits version 4.1.0^51^ (accessed October 2023), Botanical Information and Ecology Network (BIEN) version 4.2^38^ (accessed October 2023), TiP Leaf^52^ (accessed March 2023), and China Plant Trait Database v2^53^ (accessed March 2023) for the following traits, where available: vegetative height, leaf area, leaf dry mass, specific leaf area (SLA), leaf dry matter content (LDMC), leaf nitrogen (N) content per dry mass, specific root length (SRL), and seed dry mass (Table 2). Other trait databases (*e.g*., FRED^54^, GROOT^55^, LT-Brazil^56^, Tundra Traits^57^) were excluded from consideration because they were already nested within one of the databases listed above. Two traits had multiple methods of collection: (1) SLA with or without petiole included and on leaves vs leaflets and (2) SRL on all roots or fine roots only. These multiple methods of collection were included as separate traits within the dataset. Altogether, data were imputed for thirteen focal continuous traits, including these different methods of measuring the same trait (see Table 2).

Data were checked when noted (within TRY and BIEN) to ensure that all observations were taken on live plants growing under natural conditions (*e.g*., not greenhouse or growth chamber data). Within TRY, data that were noted as duplicates within the database (*i.e*., those with an Original Observation Data ID) or ranges of a trait value were removed from the dataset.

TRY continuous trait data were then filtered to remove data with Error Risk values greater than 3 (*i.e*., 3 or more standard deviations (SD) away from the mean for each trait value based on species, genera, family, or all data across the TRY database). This filtering removed 28,571 of 356,367 observations (8.0% of data). We further filtered TRY data to remove zero and negative values, which removed an additional 26 observations. Despite having removed data that were flagged within TRY as duplicates, we did find many additional cases of repeated values for some species both within and across DatasetID and ObservationID, which were filtered down to a single entry in cases where repeats could be identified as duplicate entries. Cases where it was unclear whether repeated trait values for a species were duplicate entries versus true independent measurements were left in the dataset. This filtering removed 55,650 of 327,770 observations (17.0% of data), resulting in the final inclusion of 272,120 trait records from the TRY database.

Similarly, duplicate entries within and across DatasetID and ObservationID were found within the BIEN database, which were filtered down to a single entry resulting in dropping 8,819 of 32,585 observations (27.1% of data). Further, extreme outliers in the data were checked and removed when the primary source clearly indicated that the trait was not measured in the same way as most data in the database (e.g., total leaf area for a plant rather than a single leaf), leading to an additional 2,290 of 23,766 observations being dropped (9.6% of data), resulting in the inclusion of 21,476 observations across all traits and species of interest from the BIEN database.

AusTraits, TiP Leaf, and China Plant Trait Database 2 did not contain any obviously duplicated data or extreme outliers for any species or traits of interest. Across all traits and species of interest, we included in our dataset a total of 9,673 observations from AusTraits, 2,348 from TiP Leaf, and 1,302 from China Plant Trait Database 2.

**Fig. 2:**
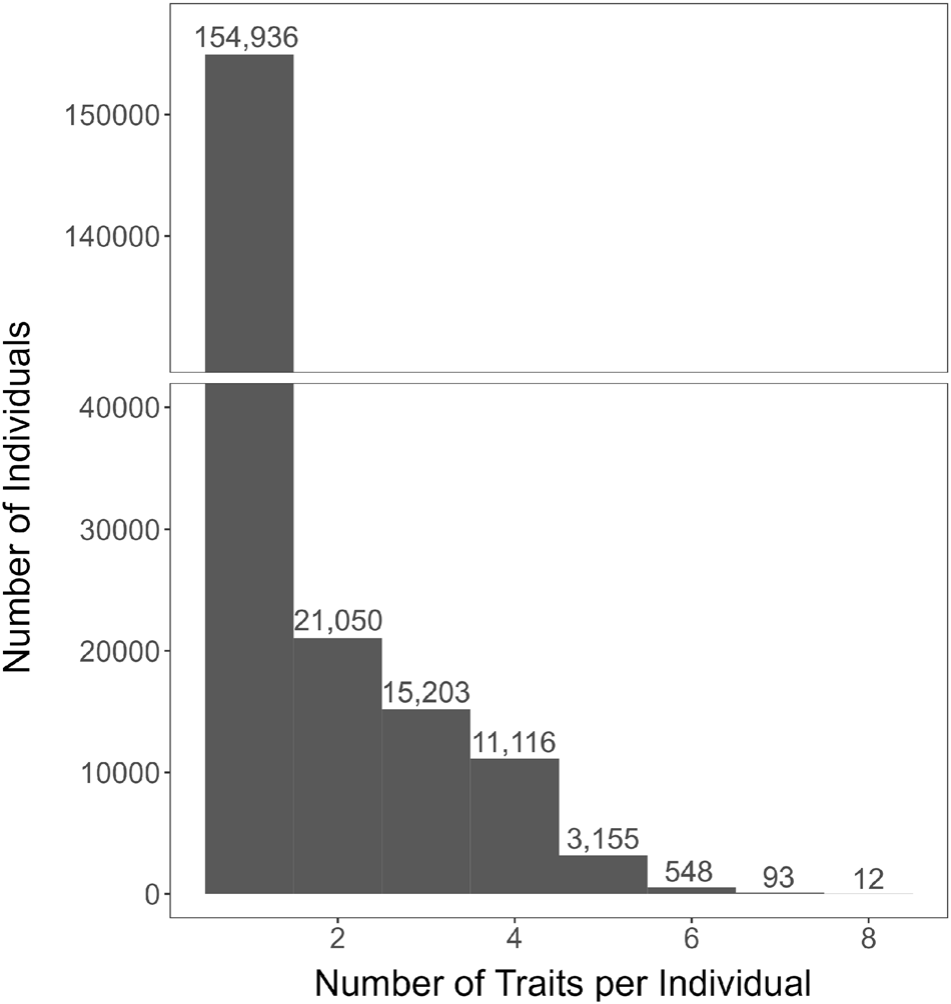
The number of observations for an individual plant ranged from one to eight focal continuous traits measured. These data served as the basis for continuous trait imputation. Numbers above the bars report the number of individual plants with the given number of traits measured. See Table 2 for a list of the continuous traits included in this dataset.

Data were then merged across all five databases (TRY, BIEN, AusTraits, TiP Leaf, and China Plant Trait Database 2), resulting in a total of 306,919 individual trait observations. This data included 206,113 plant individuals across 3,188 species in 151 families for which at least one of our thirteen focal traits (Table 2) had been measured, with 51,177 plant individuals having more than one trait measured (Fig. 2). All trait dataset and observation identifiers were retained during cleaning to allow for multiple traits that were measured on an individual plant to be linked. Units of measure were harmonized across all databases for each trait. Trait cleaning and merging code can be found in a Zenodo-archived GitHub Repository^58^ (see Code Availability below).

### Continuous trait data imputation

**Fig. 3:**
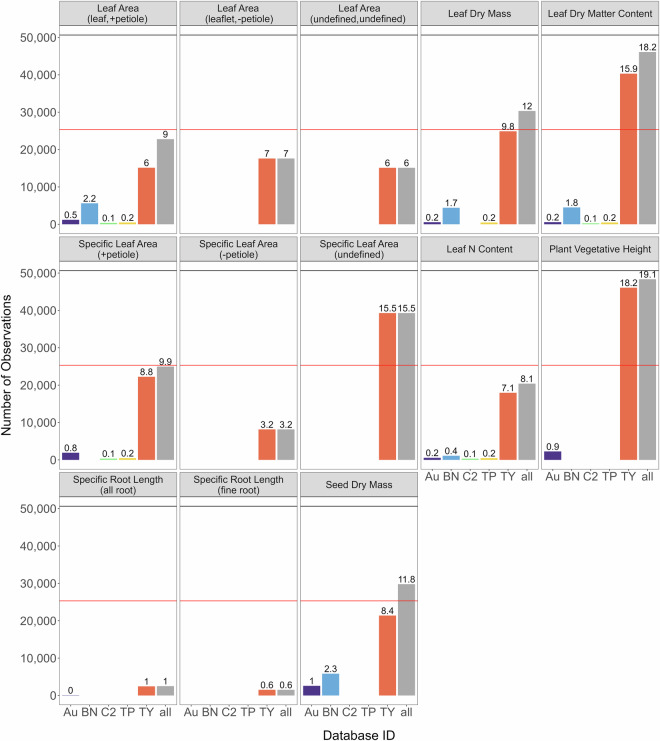
Number of observations by trait within each trait database, as well as across all databases (grey bar). The red line corresponds to 10% of trait data complete and the grey line corresponds to 20% of trait data complete for each trait. Numbers above each bar represent the percentage completeness for each trait within each trait database or across all databases. Au: AusTraits; BN: BIEN; C2: China Plant Trait Database 2; TP: TiP Leaf; TY: TRY Plant Trait Database; all: across all databases.

**Fig. 4:**
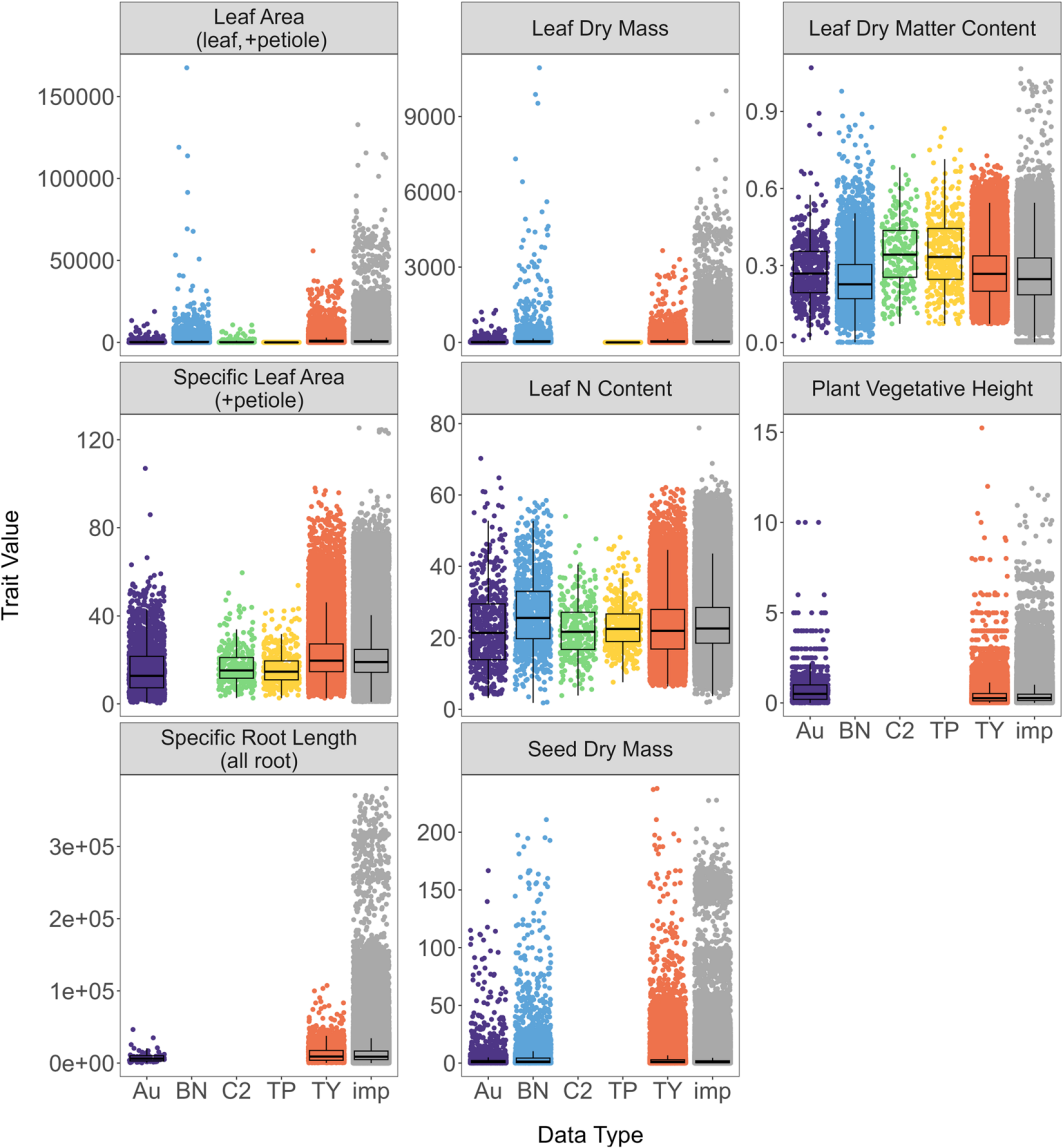
Continuous trait data from five trait databases used for trait imputation (Au, BN, C2, TP, and TRY) compared to imputed trait values (imp). Shown are mean values for each species within each trait across original and imputed data. Au: AusTraits; BN: BIEN; C2: China Plant Trait Database 2; TP: TiP Leaf; TRY: TY Plant Trait Database; imp: imputed data. See Table 2 for units.

**Fig. 5:**
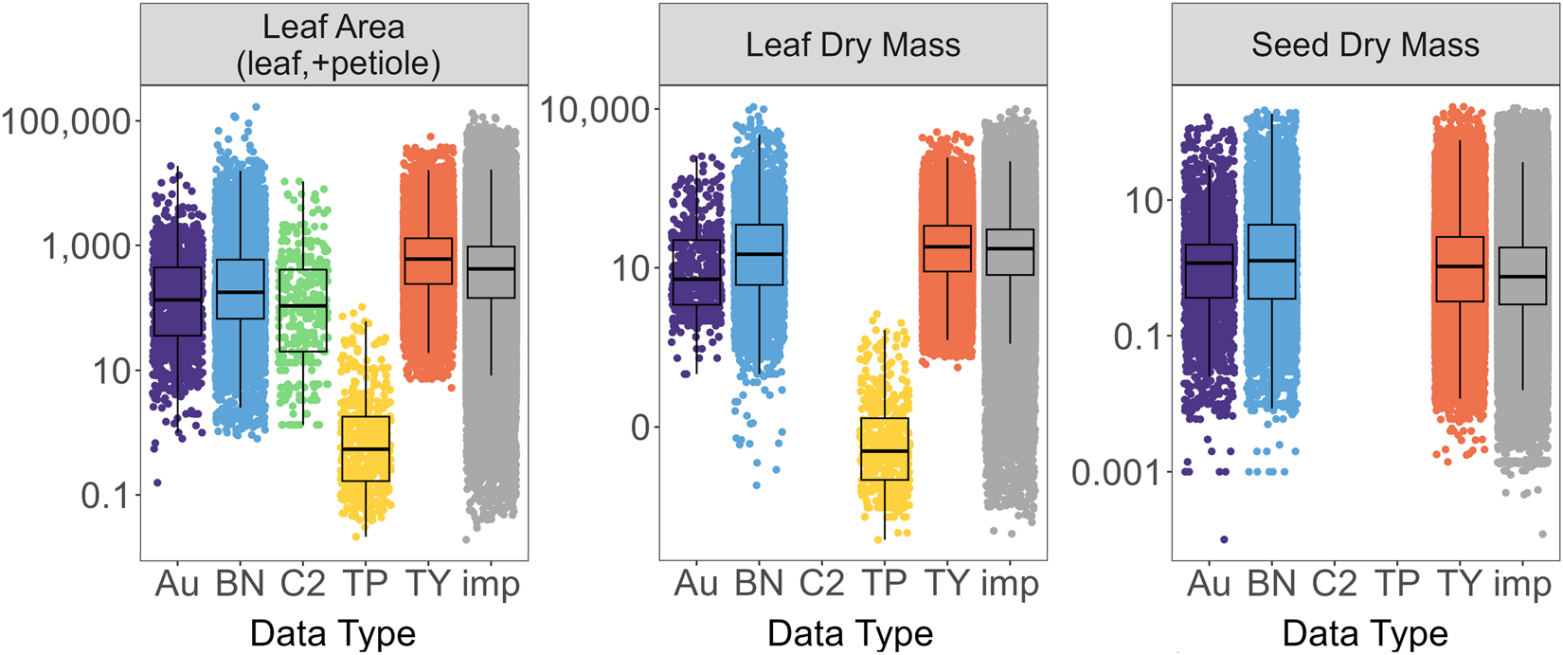
Continuous trait data for three traits plotted on a log_10_ scale for ease of visualization. Traits were derived from five trait databases used for trait imputation (Au, BN, C2, TP, and TRY) and are compared to imputed trait values (imp). Shown are mean values for each species within each trait across original and imputed data. Note that on a log_10_ scale, values between 0–1 become negative. Au: AusTraits; BN: BIEN; C2: China Plant Trait Database 2; TP: TiP Leaf; TRY: TY Plant Trait Database; imp: imputed data. See Table 2 for units.

The 306,919 observed continuous trait values were used to impute a total of 2,679,469 values in the complete dataset (88.2% missing data). Sparseness of data varied by trait (Fig. 3), with no traits that were more than 20% complete and only five traits (leaf dry mass, LDMC, SLA, vegetative height, and seed dry mass) that were at least 10% complete across all trait data. This was likely due to the lack of multiple trait measurements on any individual plant, with the majority of plant individuals only being measured for one trait (Fig. 2). Root traits were particularly sparse (Fig. 2), highlighting the need for increased investment in collection of belowground trait data. Despite the high volume of missing data, continuous traits spanned a broad range of values and were relatively consistent across databases (Figs. 4, 5). Notably, leaf area and leaf dry mass were considerably lower in the TiP Leaf database than others (Figs. 4, 5), likely because the species included in TiP Leaf^52^ are from the arid Tibetan Plateau and therefore have dry-adapted traits such as smaller leaf size.

Data were z-transformed within each trait to improve normality prior to data imputation. We then used a two-step process to first fill in missing trait values on the complete dataset and second compute species-specific averages (Fig. 1). In the first step, we employed Bayesian hierarchical probabilistic matrix factorization imputation using the R Package “BHPMF”^44^ to constrain gap-filling taxonomically. This method has previously been applied to data from the TRY database^59,60^, and has been shown to be accurate for large and sparse datasets^43^. We repeated the imputation 90 times, each time starting with different parameters (pre-fold samples = 900–1000; cross-validation steps = 10–20; burn-in steps = 10% data length). The varying parameter combinations resulted in comparable errors, as quantified by the “Root Mean Squared Error” (RMSE) falling within the range of 0.5165 to 0.5259 (mean 0.5212). Therefore, we calculated mean imputed values for each observation across all iterations. We then discarded values that were extreme (>1.5 times the maximum observed value for a trait) or uncertain (>1 SD from the mean), resulting in dropping 8,725 values (0.49% of imputed data). In the second step, we conducted five iterations of multivariate imputation by chained equations using the R package “mice”^61^ on the partially filled dataset and substituted missing cases with mean values from all iterations. Data were then back-transformed to generate the final imputed data values. Finally, we dropped five traits corresponding to multiple ways of measuring leaf area, SLA, and SRL to keep imputed data for only one method of measurement for each continuous trait (Table 2).

We calculated error risks for each trait on log_10_ transformed continuous trait values and dropped outliers with an error risk of 4 or greater across all data (*i.e*., 4 or more SD away from the mean for each trait value; 590 of 1,648,752 observations, 0.0004% of all imputed data) and within each species (8,138 additional observations, 0.005% of all imputed data). Following this data cleaning step, we calculated mean values across all observations for each species and trait combination, resulting in a final dataset^48^ of 23,410 mean imputed trait values across 2,927 species and 8 continuous traits. Note that the final number of species with imputed trait values is lower than the number of species with original data used for imputation because data cleaning steps resulted in all trait values being dropped for some species. Trait imputation code can be found in the Zenodo-archived GitHub Repository^58^ (see Code Availability below).

### Categorical trait data assembly

**Fig. 6:**
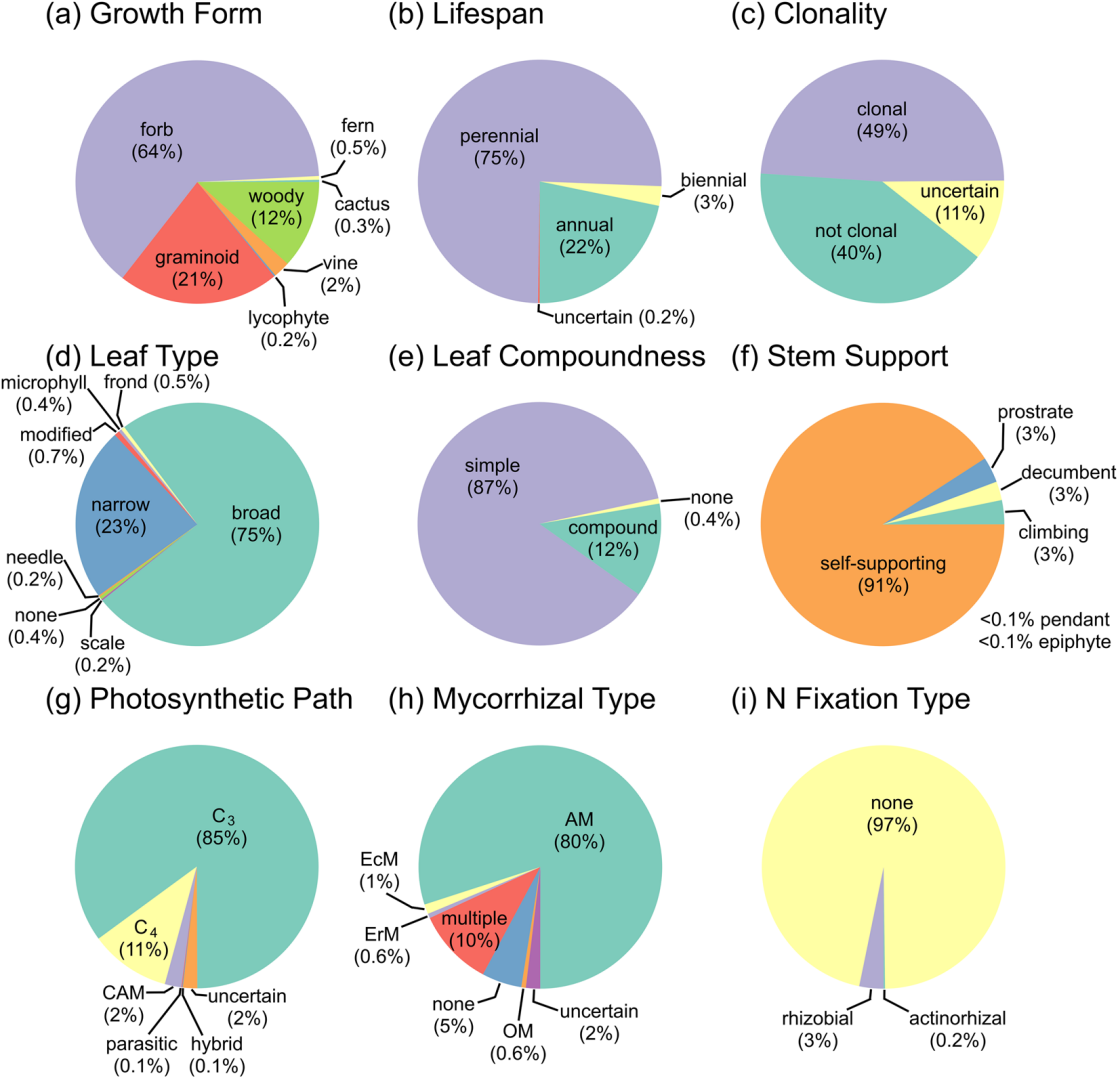
Pie charts demonstrating the frequency of occurrence of each categorical trait in the dataset. Percentages are rounded to the nearest whole number, except those <1%. Due to rounding, percentages may not add to 100% within each pie. For mycorrhizal type, AM: arbuscular mycorrhizae, EcM: ectomycorrhizae, ErM: ericaceous mycorrhizae, OM: orchidaceous mycorrhizae. Traits listed as uncertain represent those species for which the trait expression is unknown.

For each plant species in the CoRRE and GEx databases (4,079 species in 173 families), categorical trait data were collected for nine traits (Table 1; Fig. 6). Data from the TRY Plant Database were used as a starting point for all trait values except lifespan, clonality, and mycorrhizal and N fixation status. Of the 36,711 trait values needing to be filled (species by trait combinations), 9,014 values (24.6%) were obtained from TRY. For species without values for these categorical traits identified in TRY or where TRY had multiple values listed for a single species (75.4% of values), the trait value was identified through individual searches through the scientific literature, online floras, and other online resources. Additionally, we checked data from TRY for all species, with errors noted and corrected. We obtained data on mycorrhizal status from the FungalRoot Database^62^ and data on rhizobial and actinorhizal N-fixation status from the Germplasm Resources Information Network (GRIN) and Werner *et al*.^63^. Because many species have not been assessed for N-fixation status and this trait is often conserved at the genus level, we assigned all species in a genus as N-fixers for any genus that had >60% of its species as confirmed N-fixers in the dataset. For consistency across species records, leaf type and leaf compoundness data were checked for all species by K. Komatsu. Data for clonality and photosynthetic pathway were either difficult to find online or not known to science for some species. For species where clonality information was difficult to obtain, data were collected primarily by M. Avolio and R. Terry from the CLO-PLA database^64^ or evaluation of photos of herbarium root specimens. For species where photosynthetic pathway information was difficult to obtain, data were collected primarily by S. Koerner and R. Terry using information on photosynthetic pathway at the family and genus levels^65–72^ to make determinations. All other traits were divided equally among dataset authors for collection. Altogether this manual data collection took roughly 900 person hours, an impressive feat of human labour! All categorical trait records are fully referenced in the resulting dataset^48^.

## Data Records

Access to these data is available through Environmental Data Initiative (EDI). Data are being released under a CC-BY 4.0 International (CC BY 4.0) license. The BIEN data is licensed CC-BY-NC-ND, the TiP Leaf data is licensed CC-BY-NC-SA, and the FungalRoot data is licensed CC-BY-NC; however, we have been granted permission from the data owners to release this derivative under CC-BY. Any person utilizing the BIEN or TiP Leaf imputation training data or FungalRoot mycorrhizal data must comply with the original BIEN, TiP Leaf, and/or FungalRoot license terms, respectively.

The dataset^48^ contains three files: (1) CoRRE Categorical Trait Data; (2) CoRRE Continuous Trait Data; and (3) Imputation Training Data [observed trait data utilized for imputation procedures, see above for methods]. An overview of the trait definitions and units can be found in Table 1 for categorical traits and Table 2 for continuous traits.

## Technical Validation

**Fig. 7:**
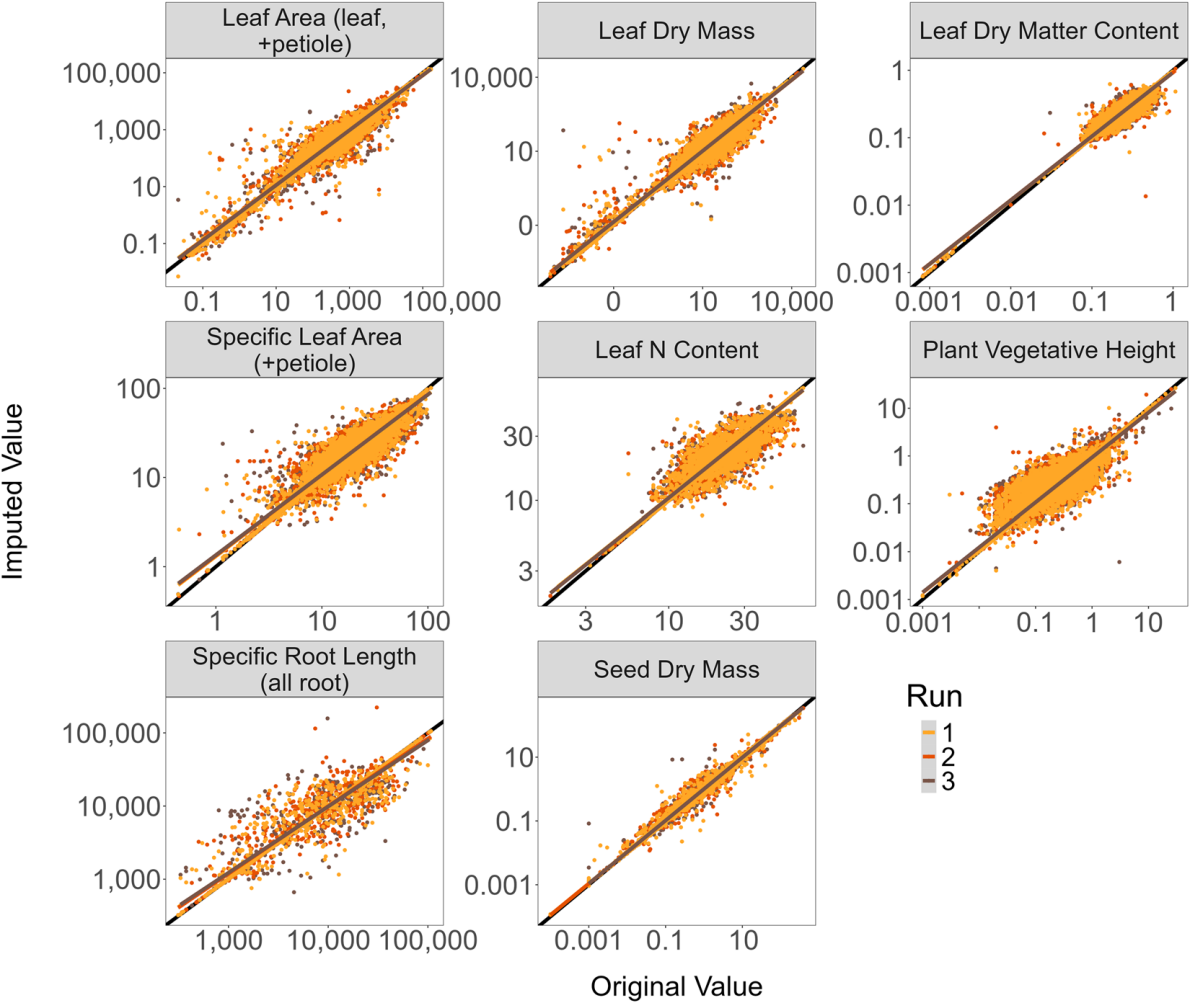
Regressions between observed and imputed values across three iterations of data validation (point and regression colors indicate validation run). The 1:1 line is shown in black. Note, all panels are plotted on a log_10_:log_10_ scale for ease of visualization. See Table 3 for fit estimates and correlation statistics for each trait.

Original trait data were split into three training datasets and used to impute the remaining trait values. Each training dataset consisted of two-thirds of the original trait data and was used to impute values for the remaining third. Training datasets were selected to preserve the underlying phylogenetic structure of the original trait data to the extent possible, with observations selected sequentially within each species and trait to be included in each training dataset. The imputed data from each validation run were then compared to the original trait data (*i.e*., data that was not part of their training datasets) to determine the accuracy of imputation of such sparse data. Training datasets each had 89.6% missing data, slightly more than our full dataset. Overall, imputed data from the validation runs were highly correlated with the original data as indicated by high Normalized Root Mean Square Error (NRMSE) demonstrating a high proportion of variance in the imputed data related to the original data and correlation coefficients (*r*) very close to 1 (Table 3; Fig. 7), lending high confidence to the use of these imputation methods for the entire dataset.

**Table 3:** Fit estimates and correlation coefficients for each of three validation runs for each trait, for each of which 2/3 of the data was used to impute the remaining 1/3.

Trait	Validation Run 1		Validation Run 2		Validation Run 3	
	NRMSE	*r*	NRMSE	*r*	NRMSE	*r*
Leaf Area	0.603	0.984	0.623	0.974	0.596	0.983
Leaf Dry Mass	0.694	0.988	0.681	0.987	0.865	0.983
Leaf Dry Matter Content	0.088	0.973	0.089	0.972	0.088	0.972
Specific Leaf Area (+petiole)	0.160	0.956	0.166	0.952	0.167	0.952
Leaf N Content	0.098	0.967	0.091	0.971	0.092	0.971
Plant Vegetative Height	0.282	0.979	0.307	0.975	0.368	0.966
Specific Root Length (all root)	0.339	0.941	0.518	0.868	0.431	0.904
Seed Dry Mass	0.309	0.996	0.300	0.996	0.306	0.996

NRMSE and correlations compared the original data (not used for training) with the imputed data for each of these runs. NRMSE: Normalized Root mean Square Error, *r*: correlation coefficient.

Mean imputed data were cleaned to drop all values with an error risk greater than 4 prior to calculating mean values of each trait for each species (see above for details). Despite this substantial data checking and cleaning effort, we caution that users of this dataset should still check that the imputed values presented here match their expectations for the species and traits they are utilizing. To aid in this effort, we present error risks (standard deviations away from the mean based on log_10_ transformed values) for each imputed value at the genus, family, and overall dataset scales. Where fewer than 3 species were present in a genus or family, the respective error risks were not calculated. Additionally, a mean of the standard deviations that were obtained from the data imputation models are included for each trait for each species to indicate which data points the imputation struggled to fit (higher values indicate less certainty). Imputed trait validation code can be found in the Zenodo-archived GitHub Repository^58^ (see Code Availability below).

For categorical trait data, 424 of the 4,079 species (10.4%) were manually checked for errors in trait entry. Of these, error rates were 0.2% for leaf type and leaf compoundness, 0.9% for growth form, 1.7% for photosynthetic pathway, 3.8% for lifespan, 3.3% for stem support, and 5.0% for clonality. Because data on mycorrhizal, rhizobial, and actinorhizal status were taken directly from other databases, their error rates were not checked beyond the values provided by the original sources^62,63^.

## Usage Notes

This Data Descriptor was peer-reviewed in June 2024 based on the CoRRE Trait Data^48^ available in EDI repository at the time. Dataset updates after June 2024 were not included in the peer-review process associated with this Data Descriptor.

We encourage users of this dataset to notify the corresponding authors if errors are identified with either incorrect categorical data or imputed continuous data that falls well outside of expectations. We intend to correct such errors in an updated version of the dataset on a yearly basis.

### Supplementary information

sCoRRE Categorical Traits Protocol

## Data Availability

All code for data processing, continuous trait imputation, and technical validation can be accessed with no restrictions through a Zenodo-archived GitHub Repository^58^ (10.5281/zenodo.11204431) and is linked to the data package in EDI. All steps were performed in R version 4.1.3.
