# Supplementary material for: CoRRE Trait Data: A dataset of 17 categorical and continuous traits for 4079 grassland species worldwide
Source: Sci Data. 2024 Jul 18;11:795. doi: 10.1038/s41597-024-03637-x (PMC11258227; doi:10.1038/s41597-024-03637-x)
Supplement: Supplementary file 1 — sCoRRE Categorical Traits Protocol [file 41597_2024_3637_MOESM1_ESM.pdf]

Supplementary File 1: Categorical Traits Protocol

Authors: K. Komatsu, M. Avolio, S. Koerner, R. Onstein, L. Korell, H. Auge, C. Roscher
Created March 27, 2020

General information: The spreadsheet contains rows for each species in the CoRRE database, with columns for each categorical trait that we are interested in for our analyses. Protocols for how to categorize species for each of the required traits are listed below. Each species can only be placed in one category for each trait (e.g., a trait cannot be listed as both annual and perennial). Trump rules are laid out below for traits where sources might give descriptions that span multiple categories.

Each trait column has a corresponding source column, where you should fill in the source used to determine the trait value for the given species (i.e., trait within a row). Ideally this would be a link to a website for a scientific paper, flora, image, etc that supports the data. If only the hardcopy of a reference is used, please list the reference as completely as possible (authors, title, date, publisher, etc).

Some trait data will already be filled in, where the information was obtained from TRY (indicated in the source column). As you are filling in the spreadsheet for your assignment, if you notice errors in the data obtained from TRY, please (a) correct them, (b) change the source in that trait's source column, and (c) add a note in the notes column with your initials indicating that you changed the TRY and for what trait.

Many species have synonyms, so if you can't find information by googling the given name, try googling the old name(s). Synonyms can be found here: http://www.worldfloraonline.org/ If you find a moss, please write "moss" in each cell of the row, no need to fill in the trait info for mosses.

The following pages contain information related to how to categorize each individual trait we are collecting.

Leaf Traits:
Leaf type, pg 3
Leaf compoundness, pg 4

Life History Traits:
Growth form, pg 5
Photosynthetic pathway, pg 5
Lifespan, pg 6
Stem-support, pg 7

Reproductive Traits:
Clonal, pg 8

Mutualisms:
Mycorrhizal type, pg 9
Nitrogen fixation type, pg 9

Additional resources: pg 10

Leaf Traits:

(a) Leaf Type (you can categorize these based on just a photo from the web)

Categories: none, broad, narrow, needle, scale, awl, frond, microphyll, modified

Definitions:

- none = no leaves, stem only (many parasitic plants)
- broad = leaves that are less than 20 times longer than they are wide; most angiosperms fall into this category (trace the outline for compound leaves)
- narrow = leaves that are more than 20 times longer than they are wide, often have sheaths; most monocots fall into this category
- needle = sharp, stiff, slender leaf of a conifer
- scale = small, thin, appressed leaf structure
- awl = awl-shape; broad at the base and tapering upward to a slender point
- frond = large, divided leaf characteristic of ferns, cycads, and palms
- microphyll = leaf with a single, unbranched leaf vein characteristic of lycophytes (aka, lycophyll)
- modified = species that have modified leaves, such as cactus (spines), carnivorous plants, etc

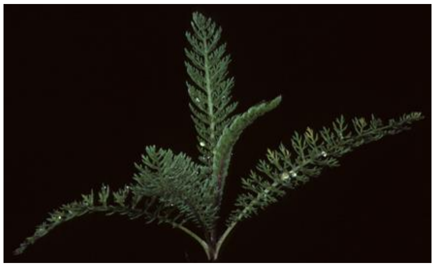

Fig. A1 (above). *Achillea millefolium* is a broad-leaf plant (highly divided, but trace the outline).

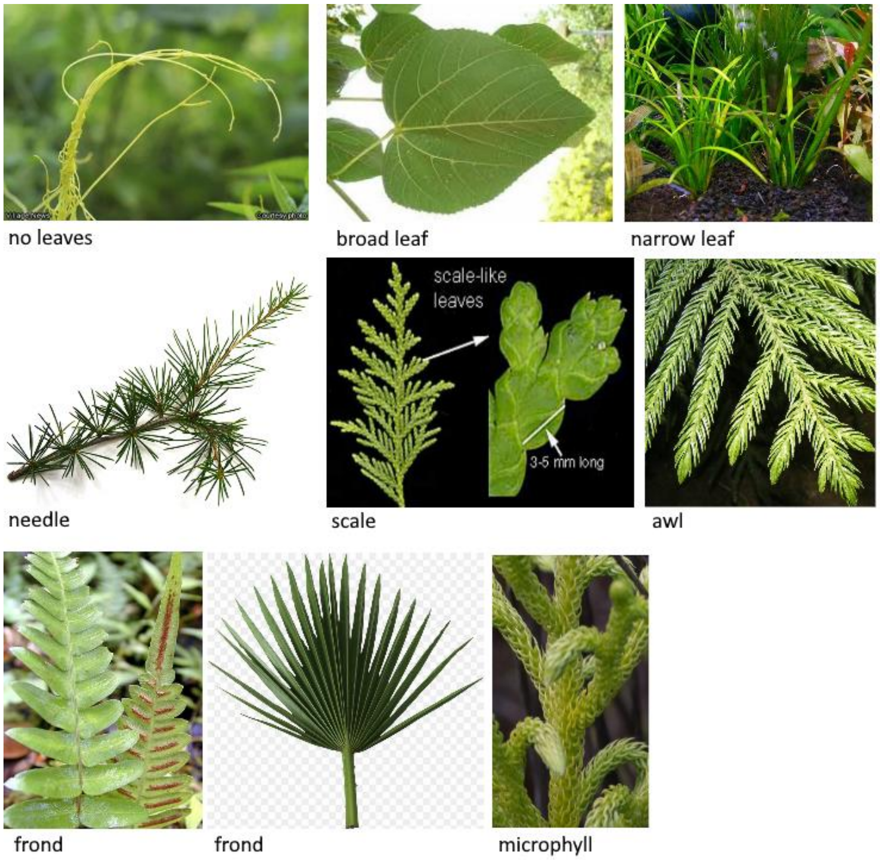

Helpful tips: For compound or highly divided leaves, consider the outline when determining shape (probably going to be broad).

Fig. A2 (left). Leaf types. (top: no leaves, broad leaves, narrow leaves; middle: needles, scales, awls; bottom: fronds, microphylls)

(b) Leaf Compoundness (you can categorize these based on just a photo from the web)

Categories: simple, compound, none

Definitions:

- simple = single leaf that is never divided into smaller leaflet units
- compound =a single leaf that is divided into smaller leaflet units; includes leaves that are singly or doubly compound, and all shapes of compoundness (e.g., palmate, pinnate)
- none = no leaves, stem only (many parasitic plants)

Trump rules:

- compound trumps simple

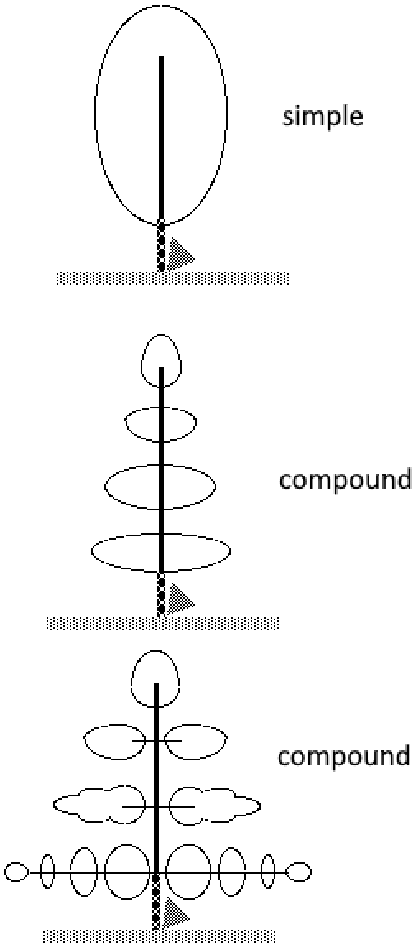

Fig. B1. Leaf compoundness.

Life History Traits:

(c) Growth Form

Categories: cactus, fern, forb, graminoid, lycophyte, vine, woody

Definitions:

- cactus = succulent plant with a thick, fleshy stem that typically bears spines and lacks leaves (Cactaceae family)
- fern = vascular plants that reproduce via spores (no seeds or flowers)
- forb = herbaceous, flowering plant other than a grass
- graminoid = grasses (Poaceae family), sedges (Cyperaceae family), rushes (Juncaceae family)
- lycophyte = fern allies, characterized by unique leaves called microphylls (Lycopodiaceae family)
- vine = plant whose stem requires support, often climbing by tendrils/twining or creeping along the ground
- woody = plants that produce wood as structural tissue (e.g., trees, shrubs); note, lianas should be classified as vines

Trump rules:

- vine trumps woody (i.e., categorize woody vines and lianas as vines)

(d) Photosynthetic Pathway

Categories: C3, C4, CAM,

Definitions:

- C3 = utilizes the C3 photosynthetic pathway
- C4 = utilizes the C4 photosynthetic pathway
- possible C4 = possibly uses C4 photosynthetic pathway (trait is characterized for the genus, but not species)
- CAM = utilizes the CAM photosynthetic pathway
- possible CAM = possibly uses CAM photosynthetic pathway (trait is characterized for the genus, but not species)
- possible C4/CAM = possibly uses C4 or CAM photosynthetic pathway (trait is characterized for the genus, but not species)
- hybrid = utilizes multiple pathways
- possible hybrid = possibly a hybrid (trait is characterized for the genus, but not species)
- parasitic = non-photosynthetic species, relies exclusively on parasitism for enery

NOTE: Only if the family has never been identified as having evolved either C4 or CAM, then it can be listed as C3. See helpful resources on pg 10.

(e) Plant Lifespan

Categories: annual, biennial, perennial

Definitions:

- annual = lifespan of less than or equal to one year
- biennial = lifespan of two years
- perennial = lifespan of more than two years

Trump rules:

- perennial trumps all other categories (e.g., if a species is listed as both an annual and a perennial, list it as a perennial)
- biennial trumps annual

(f) Stem Self-Supporting (you can categorize these based on just a photo from the web)

Categories: self-supporting, pendant, epiphyte, decumbent, prostrate, climbing

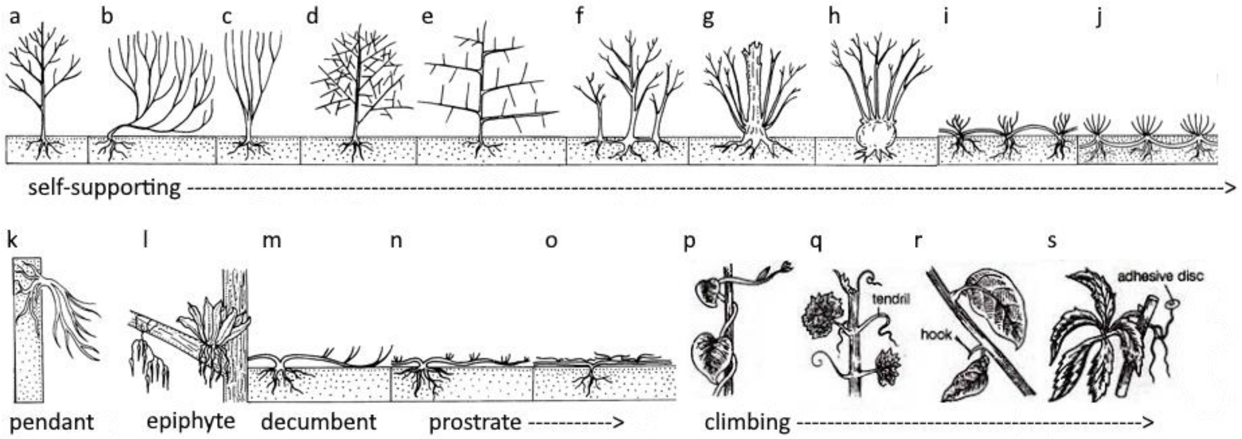

Fig F1. Examples of stem support. a-j: self-supporting. k: pendant. l: epiphyte. m: decumbent. n-o: prostrate. p-s: climbing. (modified from https://tinyurl.com/uhrt23z and https://tinyurl.com/w4ptzvj)

Definitions:

- self-supporting = stem is vertically erect and not dependent on other structures or surfaces for stature
- pendant = hanging off vertical substrate (not growing on another plant)
- epiphyte = growing entirely on another plant, but is not parasitic; not rooted in the ground
- decumbent = lying along ground/surface, with the extremity curving upward; single stem (i.e., does not set out roots along the ground)
- prostrate = lying along the ground, without curving upwards at the extremity; single stem (i.e., does not set out roots along the ground); include procumbent plants in this category
- climbing = supported by external structures/plants through the use of tendrils, twining stems, hooks, pads, etc

Trump rules:

- self-supporting always trumps all other categories (e.g., if a source describes a species as both self-supporting, decumbent, or prostrate, categorize it as self-supporting)
- climbing trumps decumbent or prostrate
- decumbent trumps prostrate
- epiphyte trumps pendant

Special cases:

(1) If a climbing species can sometimes lose its connection with the ground and become an epiphyte, categorize it as climbing

(2) If a species is referred to as clambering, scrambling, or scandent (less efficient climbers), categorize it as climbing

Reproductive Traits:

(g) Clonal

Categories: yes, no

Definitions:

- yes = clonal growth organ is either necessary for reproduction, or additive (not needed for flowering or overwintering; not present in all individuals or populations); note, **regeneration only after injury is considered non-clonal**
- no = an annual plant or not clonal under any circumstances, or only clonal following injury (regenerative clonal organ)

Helpful tips:

- Check the CloPla database (https://esajournals.onlinelibrary.wiley.com/doi/full/10.1002/ecy.1745). This table has a single entry for “clonality” (yes/no) for each species.
- Search in a web browser for the species name and “clonality” “rhizome” “stolon” and “spread” “roots” see what references say.
    - Perennials plant should be counted as clonal if:
        - they are rhizomatous or have rhizomes
        - have stolons
        - have bulbs
        - have tubers
        - are a tufted or bunch grass
    - If cuttings can be used to propagate it should not necessarily be considered clonal.
- Use photo inspection through a web browser image search.
    - If a photo shows any of the following, categorize as clonal:
        - Tussock or polycormone growth form
        - Rhizomes
        - Horizontal aboveground stems/runners
        - Vegetative buds at different plant organs either above or belowground (e.g., plantlets along the leaf margins (gemmipary) or meristems (pseudovivipary)
        - Root splitting (where senescing tap root causes fragmentation)

*****We are defining all annual plants as not clonal! Even if they can grow and spread during a growing season vegetatively, they still do not reproduce and persist by clonal means.**

Mutualisms:

(m) Mycorrhizal type

Categories: arbuscular, ecto, arbutoid, ericaceous, orchidaceous, double_AM_EcM, facultative_AM, facultative_AM_EcM, none, uncertain

NOTE: all mycorrhizal data were obtained from the FungalRoot database

Definitions:

- arbuscular = associates with arbuscular mycorrhizal fungi
- ecto = associates with ectomycorrhizal fungi
- arbutoid = associates with arbutoid mycorrhizal fungi (only species in the genera Arctostaphylos and Arbutus)
- ericaceous = associates with ericaceous mycorrhizal fungi (only ericaceous species)
- orchidaceous = associates with orchidaceous mycorrhizal fungi (only species in the family Orchidaceae)
- double_AM_EcM = associates with both arbuscular and ectomycorrhizal fungi simultaneously
- facultative_AM = chooses when it associates with arbuscular mycorrhizal fungi
- facultative_AM_EcM = chooses when it associates with either arbuscular or ectomycorrhizal fungi, but not both simultaneously
- none = never associates with any form of mycorrhizal fungi
- uncertain = uncertain from the literature which types of mycorrhizal fungi it associates with, if any

(n) Nitrogen Fixation

Categories: none, rhizobial, actinorhizal

NOTE: all nitrogen fixation data were obtained from Werner et al 2014

Definitions:

- no = never associates with any form of nitrogen-fixing bacteria
- rhizobial = associates with rhizobial bacteria in at least some circumstances
- actinorhizal = associates with actinorhizal bacteria in at least some circumstances

Helpful Resources to consult when searching for trait information

- https://plants.sc.egov.usda.gov/java/ - USDA Plants Database
- https://pladias.cz/en/ - Database of Czech flora (but has lots of species)
- https://www.wildflower.org/plants/ - Lady Bird Johnson Wildflower Center
- http://swbiodiversity.org/seinet/ - SEINet

Resources for European species:

- https://www.researchgate.net/publication/281843016_Age_of_maturity_in_alpine_herbaceous_perennials_the_North-West_Caucasus
- https://www.tandfonline.com/doi/full/10.1657/1523-0430%282005%29037%5B0602%3AEIOFAI%5D2.0.CO%3B2 (Appendix 1)
- https://clopla.butbn.cas.cz/index.php?page=gen
- https://www.jstor.org/stable/pdf/25133755.pdf?acceptTC=true&coverpage=false

Resources for United States species:

Jamex mountains plant database look at Origin starting on P416 (422/544)

Resources for Australian species:

- Part 1 Ferns, Cycads, conifers, Dicots: Acanthaceae to Asclepiadaceae
- Part 2 Dicots: Asteraceae to Buddlejaceae
- Part 3 Dicots: Cabombaceae to Eupomatiaceae
- Part 4: Dicots: Fabaceae
- Part 5 Dicots: Flacourtiaceae to Myrsinaceae
- Part 6 Dicots: Myrtaceae
- Part 7a Dicots: Nyctaginaceae to Primulaceae
- Part 7b Dicots: Proteaceae to Rubiaceae
- Part 8 Dicots: Rutaceae to Zygophyllaceae
- Part 9 Monocots: Agavaceae to Juncaginaceae
- Part 10 Monocots: Lemnaceae to Zosteraceae
- https://onlinelibrary.wiley.com/doi/10.1111/j.0906-7590.2004.04004.x (see appendix 1)

Resources for photosynthetic pathway:

- https://checklist.pensoft.net/article/19405/download/pdf/287170
- https://www.jstor.org/stable/pdf/3669716.pdf
- C4 https://academic.oup.com/jxb/article/62/9/3155/474202
- CAM: https://pdfs.semanticscholar.org/3b98/9b18355469bebabd7e1177decf7be9aebbee.pdf
- Potentially in these two paper you may be able to find your exact species or genus and learn it 100% C4, but if not, and either C4 or CAM is just possible, leave this as a "CHECK" but put in the notes that you checked and this family can be C4 or CAM depending on which paper you found it in.
- Also if you have any of these families, Amaranthaceae (Sage et al., 2007), Cleomeaceae (Marshall et al., 2007; Voznesenskaya et al., 2007; Feodorova et al., 2010), Cyperaceae (Roalson

et al., 2010), Molluginaceae (Christin et al., 2011,b), and Portulacineae (Ocampo and Columbus, 2010; Vosnesenskaya et al., 2010) consult the papers for determining C3/C4.
